# Smoking-related dysregulation of plasma circulating microRNAs: the Rotterdam study

**DOI:** 10.1186/s40246-023-00504-5

**Published:** 2023-07-10

**Authors:** Irma Karabegović, Silvana C. E. Maas, Yu Shuai, M. Arfan Ikram, Bruno Stricker, Joachim Aerts, Guy Brusselle, Lies Lahousse, Trudy Voortman, Mohsen Ghanbari

**Affiliations:** 1 Department of Epidemiology, Erasmus MC University Medical Center, Dr Molewaterplein 40, 3015 GD Rotterdam, The Netherlands; 2 Vall d’Hebron Institute of Oncology (VHIO), 08035 Barcelona, Spain; 3 Department of Pulmonary Medicine, Erasmus MC University Medical Center, Dr. Molewaterplein 40, 3015 GD Rotterdam, The Netherlands; 4 Department of Respiratory Medicine, Ghent University Hospital, 9000 Ghent, Belgium; 5 Department of Bioanalysis, Faculty of Pharmaceutical Sciences, Ghent University, 9000 Ghent, Belgium; 6 Division of Human Nutrition and Health, Wageningen University and Research, 6708 PB Wageningen, The Netherlands

**Keywords:** Circulating miRNAs, Smoking, Smoking cessation, Lung cancer

## Abstract

**Background:**

MicroRNAs (miRNAs) are post-transcriptional regulators of gene expression. Differential miRNA expression, which is widely shown to be associated with the pathogenesis of various diseases, can be influenced by lifestyle factors, including smoking. This study aimed to investigate the plasma miRNA signature of smoking habits, the potential effect of smoking cessation on miRNA levels, and relate the findings with lung cancer incidence.

**Results:**

A targeted RNA-sequencing approach measured plasma miRNA levels in 2686 participants from the population-based Rotterdam study cohort. The association between cigarette smoking (current versus never) and 591 well-expressed miRNAs was assessed via adjusted linear regression models, identifying 41 smoking-associated miRNAs that passed the Bonferroni-corrected threshold (*P* < 0.05/591 = 8.46 × 10^–5^). Moreover, we found 42 miRNAs with a significant association (*P* < 8.46 × 10^–5^) between current (reference group) and former smokers. Then, we used adjusted linear regression models to explore the effect of smoking cessation time on miRNA expression levels. The expression levels of two miRNAs were significantly different within 5 years of cessation (*P* < 0.05/41 = 1.22 × 10^–3^) from current smokers, while for cessation time between 5 and 15 years we found 19 miRNAs to be significantly different from current smokers, and finally, 38 miRNAs were significantly different after more than 15 years of cessation time (*P* < 1.22 × 10^–3^). These results imply the reversibility of the smoking effect on plasma levels of at least 38 out of the 41 smoking-miRNAs following smoking cessation. Next, we found 8 out of the 41 smoking-related miRNAs to be nominally associated (*P* < 0.05) with the incidence of lung cancer.

**Conclusions:**

This study demonstrates smoking-related dysregulation of plasma miRNAs, which might have a potential for reversibility when comparing different smoking cessation groups. The identified miRNAs are involved in several cancer-related pathways and include 8 miRNAs associated with lung cancer incidence. Our results may lay the groundwork for further investigation of miRNAs as potential mechanism linking smoking, gene expression and cancer.

**Supplementary Information:**

The online version contains supplementary material available at 10.1186/s40246-023-00504-5.

## Background

Cigarette smoking is the leading preventable risk factor responsible for a significant portion of premature deaths worldwide [1], causing about one of every five deaths in the United States each year [2]. Despite global efforts to eliminate smoking, tobacco still causes 8 million deaths each year, especially in low- and middle-income countries [3]. The link between smoking and a wide spectrum of diseases, such as cardiovascular diseases [4], chronic obstructive pulmonary disease (COPD) [5], and different types of cancer [6], is firmly established in the literature [7]. A significant number of tobacco-related fatalities are attributable to respiratory conditions such as lung cancer [8]. In this line, smoking is the leading risk factor for risk-attributable cancer burden [9] leading in the United States to almost 30% of all cancer deaths in 2019 [10]. In addition, smoking explains up to 90% in men and 70–80% in women of lung cancer risk [11], the leading cause of cancer incidence and mortality with 2.1 million new cases and 1.8 million deaths, worldwide in 2018 [12]. Although there are numerous proposed mechanisms explaining the link between tobacco use and development of lung cancer [13, 14], recent advances in omics-layers have contributed to a deeper understanding of this association [15, 16]. For instance, recent epigenomic analyses have provided a clearer understanding of the molecular pathways underpinning lung cancer development [17, 18].

Epigenetics is proposed as one of the mechanisms involved in the relationship between smoking and disease pathogenesis [19, 20], including lung cancer [17]. Epigenetics is an interface between environmental and genetic influences on disease risk, acting through gene expression, without changing the DNA sequence [21]. The major epigenetic mechanisms include DNA methylation, modification of histone proteins, and non-coding RNAs. DNA methylation is a known modulator of smoking-related alterations in cancer pathogenesis [17, 22], while non-coding RNAs have received far less attention. MicroRNAs (miRNAs) are a subset of non-coding RNAs that regulate post-transcriptional gene expression, targeting complementary messenger RNA, resulting in degradation and translational repression [23]. These small molecules are suggested to play a role in disease onset and progression [24–26] and have a potential role as biomarkers for numerous diseases [27–30], including smoking-related disorders [31].

Nevertheless, the smoking-effect on miRNA expression is not yet well-established as previous studies suffer from several limitations, including a targeted approach, limited sample size or utilizing arrays with lower coverage of miRNAs [32]. Moreover, smoking cessation is the primary prevention in reducing the risk of smoking-related diseases, such as lung cancer. Regarding miRNA expression, it is yet unknown whether the effect of smoking on miRNA expression in plasma is reversible following smoking cessation. A prior study reveals that alterations in messenger RNAs are reversible following cessation [33, 34]. However, smoking-induced dysregulation of miRNAs in small airway epithelium tissue was not reversible after quitting smoking for 3 months [35]. These contradicting results, and the short cessation time in the previous study [35], warrant the need for larger studies investigating the reversibility of the smoking-effect.

The present study aimed to investigate the smoking-related changes in plasma levels of miRNAs within the large population-based Rotterdam study cohort [36]. To this end, we tested the association between circulating miRNAs and smoking status, investigating i) current versus never smokers and ii) current versus former smokers. In addition, we explored the potential reversibility of the smoking effect following cessation by comparing current smokers with different smoking cessation time categories. Then, we examined the cumulative effect of smoking (pack-year) on miRNA expression. As smoking is the primary risk factor for lung cancer, we assessed if any of the smoking-associated miRNAs are linked to incident lung cancer in the Rotterdam study cohort.

## Results

### Study population

This study was embedded within the Rotterdam study (RS), a large prospective population-based cohort in the Netherlands [36]. Participants with smoking information and plasma miRNA levels were included in the study, resulting in 2686 independent individuals from three RS sub-cohorts (RS-I-4, RS-II-2, and RS-IV-1). Participants were categorized based on their smoking status (current, former, or never smokers), as obtained via questionnaires. The clinical characteristics, stratified by smoking status, are depicted in Table 1. In our study, 1534 (57.1%) participants were women and the mean age was 67.43 (± 11.07) years. A total of 921 (34.3%) individuals were classified as never, 1382 (51.5%) as former, and 382 (14.2%) as current smokers (Table 1).

**Table 1:** Clinical characteristics of the study population

	All sample	Never smokers	Former smokers	Current smokers
N (%)	2686	921 (34.3)	1382 (51.5)	382 (14.2)
Age (years)	67.4 (11.1)	66.3 (12.2)	68.8 (10.2)	64.6 (10.4)
Women (%)	1534 (57.1)	660 (71.6)	644 (46.6)	230 (60.2)
BMI (kg/m^2^)	27.6 (4.3)	27.66 (4.4)	27.8 (4.3)	26.8 (4.1)
Pack-year* (N = 178)	–	–	–	34.2 (31.8)

Continuous variables are expressed as mean ± standard deviation (SD), while categorical variables are expressed in numbers (percentages)

*Pack-years calculated only in current smokers, for those who had all needed data available

### Plasma miRNA levels associated with smoking habits

**Fig. 1:**
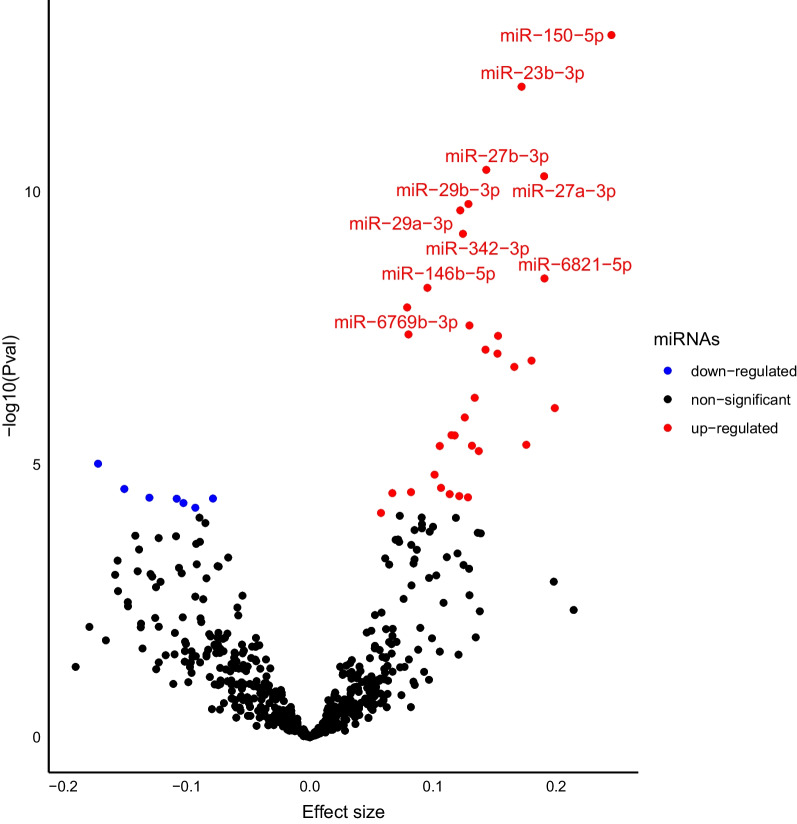
Association of plasma microRNA levels with current versus never smoking. This Volcano plot depicts the results from the linear regression model where the dots represent miRNAs tested in the association of current versus never smoking (reference) status in the Rotterdam study. The blue color depicts negatively associated miRNAs with smoking status, while the red color depicts positively associated miRNAs with smoking status. The top ten significantly associated miRNAs are annotated. The effect size per miRNA in the analysis is reflected on the X-axis while the magnitude of significance is shown on the Y-axis

Multivariable linear regression models were used to test the association between smoking (current versus never smokers [reference group]) and 591 miRNAs well-expressed in plasma (log2 CPM), adjusting for age, sex, cohort, and body mass index (BMI). A recent study showed the association between circulatory miRNAs in plasma with obesity-related traits [37] and another study showed that higher adiposity causally influences smoking behavior [38]; hence, we decided to adjust our analyses for BMI. Out of the 591 miRNAs, 41 miRNAs were differentially expressed by applying the Bonferroni-corrected significance threshold *P* < 8.46 × 10^–5^ (0.05/591) (Table 2 and Fig. 1), while 192 miRNAs were nominally associated (*P* < 0.05) (Additional file 1: Table S1). Out of the 41 smoking-miRNAs, 34 were upregulated, while 7 were downregulated in association with current vs. never smoking status (Fig. 1). The expression distributions of the significantly associated miRNAs are presented in Additional file 2: Fig. S1.

**Table 2:** MicroRNAs are significantly associated with Current versus Never (reference) smoking

*miRNA*	Beta	SE	*P-*value
*miR-150-5p*	0.2449	0.0327	1.35 × 10^–13^
*miR-23b-3p*	0.1720	0.0240	1.19 × 10^–12^
*miR-27b-3p*	0.1433	0.0215	3.98 × 10^–11^
*miR-27a-3p*	0.1903	0.0287	5.21 × 10^–11^
*miR-29b-3p*	0.1288	0.0200	1.68 × 10^–10^
*miR-29a-3p*	0.1222	0.0191	2.20 × 10^–10^
*miR-342-3p*	0.1244	0.0199	5.92 × 10^–10^
*miR-6821-5p*	0.1906	0.0321	3.89 × 10^–09^
*miR-146b-5p*	0.0956	0.0163	5.77 × 10^–09^
*miR-6769b-3p*	0.0791	0.0138	1.32 × 10^–08^
*miR-10b-5p*	0.1296	0.0232	2.83 × 10^–08^
*miR-30a-5p*	0.0802	0.0145	4.13 × 10^–08^
*miR-654-5p*	0.1529	0.0278	4.39 × 10^–08^
*miR-8069*	0.1427	0.0264	7.86 × 10^–08^
*miR-23a-3p*	0.1524	0.0284	9.28 × 10^–08^
*miR-1915-3p*	0.1801	0.0339	1.25 × 10^–07^
*miR-1237-5p*	0.1661	0.0315	1.62 × 10^–07^
*miR-149-3p*	0.1339	0.0267	5.99 × 10^–07^
*miR-4632-5p*	0.1989	0.0403	9.24 × 10^–07^
*miR-100-5p*	0.1259	0.0259	1.37 × 10^–06^
*miR-6869-5p*	0.1151	0.0245	2.89 × 10^–06^
*let-7e-5p*	0.1176	0.0250	2.93 × 10^–06^
*miR-6085*	0.1758	0.0381	4.35 × 10^–06^
*miR-30a-3p*	0.1317	0.0286	4.52 × 10^–06^
*miR-195-5p*	0.1056	0.0229	4.57 × 10^–06^
*miR-6738-5p*	0.1372	0.0301	5.66 × 10^–06^
*miR-1285-5p*	− 0.1718	0.0387	9.65 × 10^–06^
*miR-126-3p*	0.1014	0.0234	1.53 × 10^–05^
*miR-10a-5p*	0.1066	0.0253	2.68 × 10^–05^
*miR-326*	− 0.1504	0.0358	2.80 × 10^–05^
*miR-30b-5p*	0.0822	0.0197	3.21 × 10^–05^
*let-7c-5p*	0.0671	0.0161	3.34 × 10^–05^
*miR-6845-5p*	0.1136	0.0274	3.49 × 10^–05^
*miR-126-5p*	0.1214	0.0294	3.79 × 10^–05^
*miR-155-5p*	0.1285	0.0312	4.00 × 10^–05^
*miR-486-5p*	− 0.1301	0.0316	4.05 × 10^–05^
*miR-1913*	− 0.0785	0.0191	4.20 × 10^–05^
*miR-1914-5p*	− 0.1079	0.0263	4.23 × 10^–05^
*miR-93-5p*	− 0.1025	0.0252	5.07 × 10^–05^
*miR-92a-3p*	− 0.0928	0.0231	6.19 × 10^–05^
*miR-4505*	0.0579	0.0146	7.72 × 10^–05^

The table shows significant associations from the linear regression analysis where tobacco smoking was the main exposure with never-smokers as the reference group and plasma miRNA levels as the main outcome. The Bonferroni-corrected threshold was set at *P* < 0.05/591 = 8.46 × 10^–5^

When comparing current (reference group) versus former smokers, 42 miRNAs were significantly associated (*P* < 0.05/591 = 8.46 × 10^–5^), while 177 miRNAs were nominally associated (*P* < 0.05) (Additional file 1: Table S2 and Additional file 2: Fig. S2). In total, 149 miRNAs were nominally associated (*P* < 0.05) between current vs. former- and never smokers. (Additional file 1: Table S3).

### Smoking cessation and pack-years impact on plasma miRNA levels

To determine the reversibility of the smoking effect on miRNA expression levels upon smoking cessation, we calculated the time of smoking cessation in the former smokers (N = 1382) by subtracting the age at smoking cessation from the current age. This variable was further categorized into three categories; i) cessation < 5 years, ii) cessation ≥ 5 and < 15 years, and iii) ≥ 15 years of cessation. We tested the association between current smokers (reference group) and the three cessation time categories using three multivariable linear regression models, adjusting for age, sex, cohort, and BMI. Out of the 41 smoking-miRNAs that were differentially expressed between current and never smokers, 38 miRNAs had a significant difference (*P* < 0.05/41 = 1.22 × 10^–3^, and another 3 miRNAs *P* < 0.05) between current smokers and more than 15 years of smoking cessation, while 19 miRNAs (*P* < 1.22 × 10^–3^, and another 17 miRNAs *P* < 0.05) for cessation time between < 15 years and ≥ 5 (Table 3, and Additional file 2: Fig. S3). Interestingly, we show that for 2 miRNAs (*P* < 1.22 × 10^–3^, and another 7 miRNAs *P* < 0.05) there is already a significant change noticeable within only 5 years of smoking cessation, including for miR-326 and miR-6769b-3p (Table 3). The results for all 591 well-expressed miRNAs are presented in Additional file 1: Table S2 and S4 and Additional file 2: Fig. S3.

**Table 3:** Current smokers (reference) versus the cessation time categories

*miRNA*	Cessation < 5 years (N = 83)			Cessation > 5 and < 15 years (N = 161)			Cessation > 15 years (N = 535)		
	Beta	SE	*P*-value	Beta	SE	*P*-value	Beta	SE	*P*-value
*miR-326*	0.239	0.0711	**8.25 × 10**^**–04**^	0.206	0.0555	**2.23 × 10**^**–04**^	0.132	0.0396	**8.65 × 10**^**–04**^
*miR-6769b-3p*	− 0.0903	0.0275	**1.10 × 10**^**–03**^	− 0.0906	0.0218	**3.84 × 10**^**–05**^	− 0.0635	0.0148	**1.89 × 10**^**–05**^
*miR-10b-5p*	− 0.118	0.0464	1.14 × 10^–02^	− 0.194	0.0428	**7.34 × 10**^**–06**^	− 0.148	0.026	**1.65 × 10**^**–08**^
*miR-6845-5p*	− 0.134	0.0552	1.55 × 10^–02^	− 0.131	0.0425	2.24 × 10^–03^	− 0.139	0.0302	**4.70 × 10**^**–06**^
*miR-30a-3p*	− 0.14	0.0585	1.73 × 10^–02^	− 0.0845	0.0435	5.23 × 10^–02^	− 0.131	0.0322	**5.17 × 10**^**–05**^
*miR-6738-5p*	− 0.134	0.057	1.95 × 10^–02^	− 0.126	0.0422	2.92 × 10^–03^	− 0.144	0.0357	**6.16 × 10**^**–05**^
*miR-30a-5p*	− 0.0632	0.0283	2.57 × 10^–02^	− 0.101	0.0226	**1.03 × 10**^**–05**^	− 0.0855	0.0161	**1.44 × 10**^**–07**^
*miR-10a-5p*	− 0.101	0.0472	3.21 × 10^–02^	− 0.161	0.0389	**3.99 × 10**^**–05**^	− 0.125	0.0278	**7.22 × 10**^**–06**^
*miR-4505*	− 0.0608	0.0296	4.03 × 10^–02^	− 0.0812	0.0237	**6.54 × 10**^**–04**^	− 0.0425	0.0168	1.13 × 10^–02^
*miR-6821-5p*	− 0.125	0.0647	5.49 × 10^–02^	− 0.182	0.0505	**3.44 × 10**^**–04**^	− 0.174	0.0359	**1.39 × 10**^**–06**^
*miR-27a-3p*	− 0.105	0.0571	6.63 × 10^–02^	− 0.157	0.0454	**5.84 × 10**^**–04**^	− 0.173	0.0323	**1.13 × 10**^**–07**^
*miR-126-5p*	− 0.105	0.0616	8.98 × 10^–02^	− 0.17	0.0495	**6.44 × 10**^**–04**^	− 0.134	0.0359	**1.93 × 10**^**–04**^
*miR-149-3p*	− 0.0893	0.0527	9.06 × 10^–02^	− 0.122	0.0417	3.73 × 10^–03^	− 0.148	0.0297	**7.24 × 10**^**–07**^
*miR-29b-3p*	− 0.0561	0.0352	1.12 × 10^–01^	− 0.158	0.0304	**3.02 × 10**^**–07**^	− 0.125	0.0205	**1.41 × 10**^**–09**^
*miR-23a-3p*	− 0.0849	0.0547	1.21 × 10^–01^	− 0.0807	0.0436	6.50 × 10^–02^	− 0.136	0.031	**1.29 × 10**^**–05**^
*miR-195-5p*	− 0.0673	0.0455	1.40 × 10^–01^	− 0.0932	0.0356	9.18 × 10^–03^	− 0.0956	0.0258	**2.22 × 10**^**–04**^
*miR-1285-5p*	0.108	0.0754	1.53 × 10^–01^	0.0646	0.0614	2.94 × 10^–01^	0.207	0.0426	**1.37 × 10**^**–06**^
*miR-1237-5p*	− 0.091	0.0641	1.56 × 10^–01^	− 0.126	0.05	1.18 × 10^–02^	− 0.185	0.0341	**7.15 × 10**^**–08**^
*miR-1915-3p*	0.0943	0.0685	1.69 × 10^–01^	− 0.179	0.053	**7.97 × 10**^**–04**^	− 0.206	0.0366	**2.49 × 10**^**–08**^
*miR-6869-5p*	0.0712	0.0519	1.71 × 10^–01^	− 0.0806	0.0403	4.61 × 10^–02^	− 0.134	0.0294	**6.06 × 10**^**–06**^
*miR-8069*	0.0768	0.0614	2.11 × 10^–01^	− 0.116	0.0481	1.59 × 10^–02^	− 0.126	0.0313	**6.25 × 10**^**–05**^
*miR-6085*	0.0996	0.0796	2.12 × 10^–01^	− 0.226	0.0616	**2.72 × 10**^**–04**^	− 0.212	0.0425	**7.59 × 10**^**–07**^
*miR-93-5p*	0.0629	0.0517	2.24 × 10^–01^	0.105	0.0401	9.25 × 10^–03^	0.0949	0.0293	1.25 × 10^–03^
*miR-654-5p*	0.0786	0.0658	2.32 × 10^–01^	− 0.14	0.0441	1.55 × 10^–03^	− 0.135	0.0324	**3.39 × 10**^**–05**^
*miR-27b-3p*	0.0486	0.041	2.36 × 10^–01^	− 0.159	0.0343	**4.76 × 10**^**–06**^	− 0.157	0.0233	**3.00 × 10**^**–11**^
*miR-1913*	0.0425	0.0359	2.37 × 10^–01^	0.0776	0.0276	5.20 × 10^–03^	0.059	0.0202	3.57 × 10^–03^
*miR-126-3p*	− 0.0543	0.0466	2.45 × 10^–01^	− 0.124	0.0367	**7.85 × 10**^**–04**^	− 0.107	0.0259	**4.02 × 10**^**–05**^
*miR-1914-5p*	0.0605	0.0534	2.57 × 10^–01^	0.0766	0.0406	5.96 × 10^–02^	0.0985	0.0288	**6.47 × 10**^**–04**^
*miR-23b-3p*	− 0.0538	0.0478	2.61 × 10^–01^	− 0.128	0.0385	**9.11 × 10**^**–04**^	− 0.154	0.0259	**3.83 × 10**^**–09**^
*miR-150-5p*	− 0.0796	0.0709	2.62 × 10^–01^	− 0.231	0.0543	**2.48 × 10**^**–05**^	− 0.203	0.0365	**3.60 × 10**^**–08**^
*miR-100-5p*	− 0.0541	0.0511	2.90 × 10^–01^	− 0.178	0.0416	**2.09 × 10**^**–05**^	− 0.135	0.0286	**3.02 × 10**^**–06**^
*miR-29a-3p*	− 0.0372	0.0392	3.44 × 10^–01^	− 0.134	0.0317	**2.82 × 10**^**–05**^	− 0.116	0.0207	**3.37 × 10**^**–08**^
*let-7e-5p*	0.0332	0.0464	4.74 × 10^–01^	− 0.0874	0.0383	2.28 × 10^–02^	− 0.125	0.0275	**6.26 × 10**^**–06**^
*miR-146b-5p*	− 0.015	0.0314	6.34 × 10^–01^	− 0.0503	0.0252	4.67 × 10^–02^	− 0.0741	0.018	**4.14 × 10**^**–05**^
*miR-342-3p*	− 0.0174	0.0388	6.55 × 10^–01^	− 0.102	0.0314	1.24 × 10^–03^	− 0.125	0.0249	**5.89 × 10**^**–07**^
*miR-30b-5p*	0.0134	0.0369	7.16 × 10^–01^	− 0.0796	0.0306	9.68 × 10^–03^	− 0.0723	0.0212	**6.81 × 10**^**–04**^
*miR-92a-3p*	0.0156	0.0457	7.34 × 10^–01^	0.0809	0.0361	2.53 × 10^–02^	0.0917	0.0266	**5.93 × 10**^**–04**^
*let-7c-5p*	0.00796	0.0302	7.92 × 10^–01^	− 0.0885	0.0265	**8.98 × 10**^**–04**^	− 0.0663	0.0177	**1.87 × 10**^**–04**^
*miR-4632-5p*	− 0.00905	0.0820	9.12 × 10^–01^	− 0.125	0.0641	5.18 × 10^–02^	− 0.18	0.0457	**8.56 × 10**^**–05**^
*miR-155-5p*	− 0.00327	0.0596	9.56 × 10^–01^	− 0.15	0.0488	2.20 × 10^–03^	− 0.121	0.035	**5.77 × 10**^**–04**^
*miR-486-5p*	0.00327	0.0638	9.59 × 10^–01^	0.16	0.0495	1.32 × 10^–03^	0.154	0.0366	**2.79 × 10**^**–05**^

The table shows for the smoking-miRNAs the results from the linear regression analysis where tobacco smoking was the main exposure with current smokers as the reference group versus the different smoking cessation categories, and plasma miRNA levels as the main outcome. The Bonferroni-corrected threshold was set at *P* < 0.05/41 = 1.22 × 10^–3^. The *p*-values presented in bold passed the Bonferroni-corrected threshold. SE: standard error

Next, we investigated the association between the cumulative effect of smoking in current smokers (pack-year) as exposure and all miRNA levels as the outcome. Pack-years was calculated in all current smokers; as the number of cigarettes smoked per day, divided by 20, and multiplied by the total years of smoking. None of the miRNAs passed the Bonferroni-corrected threshold (*P* < 8.46 × 10^–5^), in association with pack-years in current smokers (n = 178), while 14 miRNAs were nominal significant (*P* < 0.05) (Additional file 1: Table S5).

### Association of smoking-related miRNAs and incident lung cancer

As smoking is the main modifiable risk factor for lung cancer, we tested if the identified smoking-miRNAs are associated with incidence of lung cancer in an exploratory analysis. We used a subset of 1806 participants for which we had data available and that were free of lung cancer at the moment of miRNA quantification, while the clinical characteristics are presented in Additional file 1: Table S6. During a mean follow-up period of 8.8 (± 3.1) years, 37 participants developed lung cancer. The Cox proportional hazards regression was used to determine the hazard ratios (HRs) and 95% confidence intervals (CIs) between miRNA expression and incident lung cancer, adjusted for age, sex, cohort, smoking, chronic diseases, BMI, red blood cells (RBC), white blood cells (WBC), alcohol consumption, and education. Out of the 41 smoking-related miRNAs, we identified eight miRNAs related to lung cancer (*P* < 0.05), including miR-146b-5p, miR-6769b-3p, miR-1915-3p, miR-6085, miR-10a-5p, miR-100-5p, miR-149-3p, and let-7c-5p (Table 4 and Additional file 1: Table S7). Out of these, six miRNAs suggest an increased risk for lung cancer and two show a protective effect (Table 4).

**Table 4:** Association of smoking-related miRNAs with incident lung cancer

miRNA	HR	95% CI	*P*-value
*miR-1915-3p*	0.47	0.24–0.90	0.024
*miR-146b-5p*	6.1	1.48–25.14	0.012
*miR-6085*	0.56	0.33–0.94	0.030
*miR-6769b-3p*	5.61	1.20–26.18	0.028
*miR-10a-5p*	2.67	1.29–5.52	0.008
*let-7c-5p*	3.93	1.19–12.94	0.024
*miR-100-5p*	2.22	1.19–4.12	0.012
*miR-149-3p*	1.58	1.08–2.31	0.018

This table shows the results for the Cox proportional hazards regression adjusted for age, sex, cohort, smoking, chronic disease, BMI, RBC, WBC, alcohol, and education

Hazard ratios (HRs) are reported for each log2 unit increase in miRNA level in the longitudinal setting

### In silico analysis of target genes of smoking-related miRNAs

We examined whether predicted target genes of the smoking-related miRNAs are associated with smoking status. Focusing only on unique target genes reported by three (TargetScan [39], miRDB [40], and miRTarBase [41]) miRNA target gene prediction platforms, we extracted 407 predicted target genes for the 41 smoking-associated miRNAs (Additional file 1: Table S8). Seven target genes were identified to be associated with smoking in a genome-wide association study (GWAS) for smoking initiation [42], 140 genes have an annotated CpG that was identified in an epigenome-wide association study (EWAS) on smoking habits [43], while a single gene (*CADM1*) was identified in a transcriptome-wide association study (TWAS) on smoking trait (cigarettes/day) (Additional file 1: Table S9). Using miRPathDB 2.0 [44], we explored the KEGG pathways [45] for the identified miRNAs and observed that many of them were implicated in several cancer pathways (Additional file 2: Fig. S4). In addition, out of the eight miRNAs we identified to be linked with lung cancer, two (miR-146b-5p and let-7c-5p) showed to be implicated in several cancer-related pathways, and miR-146b-5p in both small cell lung cancer and non-small cell lung cancer.

## Discussion

In this study, we investigated the association between smoking habits and 591 miRNAs well-expressed in plasma in a population-based cohort study. In total, 41 miRNAs were significantly associated with current versus never (reference) smoking after adjustment for multiple testing. Moreover, 42 miRNAs were significantly associated with current (reference) versus former smokers. While testing the reversibility of the smoking effect on miRNA levels, we compared miRNAs levels in current smokers (reference) with those of three cessation time categories. For 38 out of the 41 smoking-miRNAs there was a significant difference between current smokers and after more than 15 years of smoking cessation. Finally, we found eight of the 41 smoking-related miRNAs to be associated with incident lung cancer.

An increasing number of studies have investigated smoking with miRNA expression levels [32, 46, 47]. However, these studies were mainly conducted either on a subset of miRNAs using a qPCR-based method or in a modest sample size [32]. Despite this lack of method consensus, we validated some of the findings in previous studies. For instance, in a large-scale study (whole blood qPCR assessed miRNAs, n = 5000) by Willinger et al. [47], 6 miRNAs (out of 283) were linked to smoking, of which miR-1285-5p and miR-342-3p, arising from the same precursors, were replicated in our study [47]. In addition, miRNAs we have identified in this study (miR-126 and miR-195) were previously linked to smoking in small airway epithelium [35]. Some studies have demonstrated that the mature miRNA sequences from the 5’ and 3’ arms of the precursor duplex (assigned -5p or -3p) are often co-expressed [48, 49]. In this line, plasma level comparison of qPCR-quantified miRNAs in smokers (n = 11) and non-smokers (n = 7) identified 43 differently expressed miRNAs [46], of which eight pre-miRNAs overlap with our findings. Although other studies on smoking and miRNAs reported different methodologies and/or tissue, which makes reproducibility between cohorts challenging, we were able to validate some of their findings [35, 46, 47].

To the best of our knowledge, this paper is the first to explore, in addition to identifying smoking-related miRNAs utilizing novel next-generation sequencing (NGS) platform for a broad landscape of miRNAs, the potential for reversibility of the changes in plasma miRNA levels. In this study, we identified the potential for reversibility of smoking-related changes in plasma miRNA levels by comparing current smokers (reference group) in relation to former smokers at a population-based level. We found significant differences between current smokers and the length of smoking cessation, where smokers overall have higher miRNA levels than non-smokers and that these levels seem to lower with longer smoking cessation time. These findings may indicate that smoking-related changes in plasma miRNA levels are reversible upon smoking cessation. This conjecture is in line with previous studies on the reversibility of changes in gene expression (messenger-RNAs) following smoking cessation [33, 34]. Nevertheless, future research is needed to test these findings with repeated measurements.

Many of the miRNAs we have identified herein have previously been linked to smoking-related diseases [50–53]. As example, previous studies have demonstrated our top finding miR-150-5p to be associated with chronic obstructive pulmonary disease (COPD) [50, 51]. Interestingly, miR-150-5p is frequently deregulated in cancer and regulates the gene expression of several cancer driver genes, including in smoking-related cancers, such as lung cancer and colorectal cancer, and in non-smoking related cancer types [2], such as breast and prostate cancer [54–60]. Furthermore, newly identified smoking miR-27a-3p was previously linked with lung cancer and COPD [52]. Molina-Pinelo et al. [53] also identified three of smoking-related miRNAs identified herein (miR-486-5p, miR-146b-5p, and miR-342-3p) to be associated with COPD and/or lung adenocarcinoma. Notably, we also identified miR-146b-5p in association with incident lung cancer. Our results may endorse previous literature that respiratory diseases share common risk factors of smoking, potentially through pathways regulated partly by epigenetic mechanism including some of the miRNAs identified herein. In addition, our investigation of the cumulative effect of smoking (measured by pack-years) on miRNA levels did not reveal any significant associations. Nevertheless, these results might have been hindered by the limited sample size (n = 178).

Lastly, we investigated the putative target genes of smoking-associated miRNAs and identified links with smoking through previous (epi-)genetic and transcriptomic studies, including GWAS, epigenome- and transcriptome-wide association studies [42, 43, 61]. In addition, some of the identified smoking-related miRNAs are implicated in cancer pathways. These findings may suggest the potential of identified miRNAs to partially explain the link between smoking and lung cancer, which warrant confirmation by experimental studies in future.

The major strengths of the study presented, are the use of data from a large-scale population-based cohort and the measurement of miRNA levels via a specific, sensitive, and reproducible targeted RNA-sequencing method [62]. Nevertheless, the findings presented in this manuscript should be interpreted with caution. Our results need to be further replicated in an independent cohort. Though part of our results show overlap with previous studies, despite using different methodologies and/or tissues for quantifying miRNA levels. In addition, as miRNAs are tissue-specific, perhaps other tissues such as lung, would provide a better setting to confirm the smoking effect on miRNA levels linked to lung cancer. In addition, although we adjusted our models for possible confounding effects, we cannot exclude the possibility of residual confounding of smoking, as the passive smoking or smoking of other tobacco products (e.g., cigars, hookahs, pipes, and cannabis) are not captured by the questionnaires. Furthermore, individuals' answers on smoking behaviour might be impacted by social desirability bias. In line with this, it is hard to decipher what constitutes someone as former smoker, given the collection of data presented herein, which might have impacted our analysis on former smokers. Moreover, in-depth information regarding environmental or occupational exposures that can possibly affect miRNA expression, such as air pollution and asbestos, were not available for model adjustments. Further experimental validation is warranted to assess the impact of smoking on the identified miRNAs and the subsequent risk of disease attributable to smoking.

## Conclusion

In summary, we present a large-scale population-based investigation of plasma circulating miRNAs in relation to cigarette smoking. The evidence from this study suggests multiple miRNAs to be associated with cigarette smoking, many of them seem to be reversible following smoking cessation. In addition, we provide evidence for potential correlations between some of the smoking-related miRNAs and incident lung cancer. These results may lay the groundwork for further investigation of miRNAs as epigenetic modulators linking smoking, gene expression, and lung cancer.

## Methods

### Study population

This study was conducted using data from the RS, a large prospective population-based cohort initiated in 1989 in the city of Rotterdam, the Netherlands [36]. The RS has four sub-cohorts, and participants (> 40) are followed every 3–5 years. The first sub-cohort (RS-I) includes 7983 individuals (age ≥ 55 years). This was later extended by a second sub-cohort (RS-II, n = 3011, age ≥ 55 years), and a third sub-cohort (RS-III, n = 3932, age ≥ 45 years). The most recent extension includes 3005 individuals, as the fourth sub-cohort (RS-IV, age ≥ 40 years). A more in-depth description of the RS can be found elsewhere [36]. The RS has been approved by the Medical Ethics Committee of the Erasmus MC and by the review board of the Dutch Ministry of Health, Welfare and Sports (1,068,889–159,521-PG).

Plasma levels of circulating miRNAs were measured in 2754 randomly selected individuals from three RS sub-cohorts (RS-I-4, RS-II-2, and RS-IV-1). One individual was excluded due to missing profiling on miRNAs, while 63 participants were excluded due to missing smoking data, resulting in a sample size of 2686 non-overlapping participants.

### MicroRNA profiling

The HTG EdgeSeq miRNA Whole Transcriptome Assay (WTA) was used to measure the levels of miRNAs in plasma. Whole blood samples in Rotterdam study were collected in PAXGene Tubes. A total volume of 50 μL of plasma, for two re-measurements that generally is sufficient to obtain a valid result for all samples, was sent to HTG Molecular Diagnostics, Inc. (AZ, USA) for sequencing. Each sample was tagged individually with molecular barcodes; tagged samples were pooled and sequenced on an Illumina NextSeq sequencer (Illumina, San Diego, CA, USA). Data were provided as data tables of raw, quality control (QC) raw, counts per million (CPM), and median normalized counts. Log2 counts per million (log2 CPM) standardization was used to transformed counts and adjusted for total reads within a sample. The initial miRNA list encompassed all 2083 miRNAs in the HTG EdgeSeq miRNA Whole Transcriptome Assay that were profiled in 2754 Rotterdam study participants. miRNAs with Lg2 CPM < 1.0 indicated that they were not expressed in the samples. We implemented a lower limit of quantification (LLOQ) method to select well-expressed miRNAs. The LLOQ level was based on a monotonic decreasing spline curve fit (by R function ‘scam::scam’) between the mean and standard deviation per miRNA on the normalized value. All miRNAs of which > 50% of the values were above the LLOQ were considered as well-expressed (n = 591).

### Smoking and lung cancer assessment

Participants were categorized into smoking status categories (former, current, and never) based on the answers they provided in self-administered questionnaires. In former smokers, smoking cessation was calculated based on the age minus the cessation age. Due to the low response rate on “cessation age” cross-sectionally, we used the previous time-point for former smokers in both time points (i.e., did not initiate smoking meanwhile). This variable was further categorized into i) cessation less than 5 years ago (< 5 years), ii) between 5 and 15 years (≥ 5 and < 15 years), and iii) more than 15 years (≥ 15 years).

Additionally, for the cumulative effect of smoking on miRNA levels in current smokers, we computed pack-years (number of cigarettes smoked per day, divided by 20, multiplied by the total years of smoking). The smoking initiation age was not available in the cross-sectional setting but was used from the previous visit for a subset of cohorts, which had available data. We calculated pack-year for current smokers at both points. One participant was excluded due to initiation at the age of five, which we considered an outlier.

Lung cancer was diagnosed from the general practitioner's medical records and through linkage with Dutch Hospital Data, Netherlands Cancer Registry, and histology and cytopathology registries. Two physicians independently coded diagnoses according to the International Classification of Diseases, tenth revision (ICD-10). In case of discrepancy, a consensus was sought through physician specialized in internal medicine. Due to small sample size, histological lung cancer diagnosis included all lung cancer types. Lung cancer diagnosis date was based on the biopsy date; if unavailable, the hospital admission date, or using the discharge letter. Only pathology-confirmed lung cancers were included in the analysis. The follow-up for incident lung cancer was conducted until January 1, 2015. Participants were followed from study entry until the occurrence of cancer, death, the last health status update when they were known to be cancer-free, or January 1, 2015, whichever came first. Incident lung cancer was defined as any primary lung cancer. In the case of multiple cancers within one participant, we included only those whose first diagnosis was lung cancer for analysis, while the rest were excluded.

### Covariable assessment

Home-administered interviews were used to assess participants’ age and sex. Weight and height were measured when participants were standing without heavy outer garments or shoes. Information on weight and height was used to calculate participants' BMI as weight divided by height squared (kg/m^2^). Educational level (primary, lower, intermediate, and higher) and alcohol consumption (g/day) were assessed during the home interviews. Blood pressure (BP) was measured twice in a sitting position on the right arm using a random-zero sphygmomanometer, and the average of 2 measurements was used. Hypertension was defined as a systolic (BP) ≥ 140 mm Hg or diastolic BP ≥ 90 mm Hg or the use of BP‐lowering drugs prescribed for hypertension. Prevalent diabetes mellitus type 2 was identified according to the World Health Organization criteria: fasting glucose levels of ≥ 7.0 mmol/L, nonfasting glucose levels ≥ 11.1 mmol/L, or the use of glucose-lowering medication. Coronary heart disease was defined if the participant suffered a myocardial infarction or underwent a coronary artery bypass grafting or percutaneous coronary revascularization procedure. Stroke was defined according to the World Health Organization definition as a syndrome of rapidly developing clinical signs of focal or global disturbance of cerebral function, with symptoms lasting 24 h or longer or leading to death, with no apparent cause other than of vascular origin. Participants were screened for dementia at baseline and subsequent center visits with the Mini-Mental State Examination and the Geriatric Mental Schedule organic level [63]. Those with a Mini-Mental State Examination score < 26 or Geriatric Mental Schedule score > 0 underwent further investigation and informant interview, including the Cambridge Examination for Mental Disorders of the Elderly. Blood samples of participants were obtained during the visit to the research centre. Using a haematology analyser, measure the levels of red blood cell counts (1012/L) and white blood cell counts in venous blood (109/L).

### Statistical analyses

#### Smoking in association with changes in plasma miRNA levels

We implemented multivariable linear regression models to explore the association between smoking (current versus never smokers [reference group]) and plasma miRNA levels (log2 CPM), adjusted for age, sex, cohort, and BMI. We used the same adjustment for other smoking-exposure analyses throughout this manuscript, and the Bonferroni-corrected P-value threshold was set at *P* < 0.5/591 = 8.46 × 10^–5^. We also explored current (reference group) versus former smokers with miRNA expression levels. Next, we assessed the potential of reversibility of the smoking effect by comparing expression levels of the identified smoking miRNAs among different cut-off groups within former smokers. We compared the difference in miRNA levels between current smokers (reference) with the three cessation time categories, including i) < 5 years, ii) ≥ 5 and < 15 years, and iii) ≥ 15 years of cessation time. Finally, we investigated the association between the cumulative effect of smoking in current smokers (pack-year) as exposure and all miRNA levels as the outcome.

Additionally, we explored the relationship between smoking-related miRNAs and the incidence of lung cancer. Due to the presence of competing mortality risks, we applied the competing risk Cox proportional hazards regression model to determine hazard ratios (HRs) and 95% CIs between miRNA expression and incident lung cancer. The analyses were adjusted for age, sex, cohort, smoking, chronic disease, BMI, red blood cells, white blood cells, alcohol consumption, and education. Nominal P-value threshold (*P* < 0.05) was considered, due to the correlation between the pre-selected smoking-miRNAs and the exploratory nature of our analysis. All analysis were performed using R software, version 4.2.3 (R Core Team, 2021).

#### In silico analyses of target genes of smoking-associated miRNAs

We used the open-source platform miRWalk [64] to obtain putative and validated miRNA target genes. Our selection criteria were based on genes that were reported in all three commonly used miRNA prediction databases (TargetScan [39], miRDB [40], and miRTarBase [41]), embedded within the miRWalk platform [64]. We included genes which were reported in all three databases. We explored if the miRNA predictive target genes were previously linked to smoking through GWAS [42], EWAS [43], and TWAS studies [61]. Using miRPathDB 2.0 [44], we explored the KEGG pathways [45] underlying the smoking-related miRNAs.

## Supplementary Information

**Additional file 1**: **Table S1**. Provides the results for the multivariable linear regression analysis between current and never smokers; **Table S2**. Provides the results for the multivariable linear regression analysis between currentand former smokers; **Table S3**. Provides an overview of the miRNAs that are nominally associated in both current versus never and current versus former; **Table S4**. Provides the results for the multivariable linear regression analysis between currentand cessation time categories; **Table S5**. Provides the results for the linear regression analysis on cumulative effect of smoking; **Table S6**. Provides the clinical characteristics for the sample population in the lung cancer exploratory analysis; **Table S7**. Shows the results for the exploratory lung cancer incidence analysis; **Table S8**. Provides the list of target genes for the 41 smoking-miRNAs, and **Table S9**. Shows which target genes were previously identified in association with smoking status in GWAS, EWAS, or TWAS.

**Additional file 2**: **Fig. S1**. Provides the distribution of the top 10 significantly associated miRNAs with current versus never smoking status; **Fig. S2**. Provides a Volcano plot depicting the results from current versus former smokers analysis, where current smoking was a reference; **Fig. S3**. Depicts boxplots of all 41 smoking miRNAs with their expression levels across different smoking cessation categories, while **Fig. S4.** Depicts the enrichment plot for the smoking associated miRNAs in the KEGG pathways.

## Data Availability

The Rotterdam Study data can be made available to interested researchers upon request. Requests can be directed to data manager Frank J. A. van Rooij (f.vanrooij@erasmusmc.nl) or visit the following website for more information: https://www.ergo-onderzoek.nl/contact. We are unable to place data in a public repository due to the confidential nature of the data collected and legal and ethical constraints.

## Abbreviations

Beta: Effect size estimate
BMI: Body mass index
BP: Blood pressure
CI: Confidence interval
COPD: Chronic obstructive pulmonary disease
EWAS: Epigenome-wide association study
GWAS: Genome-wide association study
HR: Hazard ratio
MiRNAs: MicroRNAs
NGS: Next-generation sequencing
RBC: Red blood cells
RS: Rotterdam study
SE: Standard error
TWAS: Transcriptome-wide association study
WBC: White blood cells

## References

[1] Bergen AW, Caporaso N (1999). Cigarette smoking. J Natl Cancer Inst.

[2] Prevention CfDCa. 2014 Surgeon general’s report: The health consequences of smoking—50 years of progress: centers for disease control and prevention; 2014 [Available from: https://www.cdc.gov/tobacco/data_statistics/sgr/50th-anniversary/index.htm. 24455788

[3] Organization WH. Tobacco: WHO; 2021 [updated 26/07/2021. Available from: https://www.who.int/news-room/fact-sheets/detail/tobacco.

[4] McBride PE (1992). The health consequences of smoking. Cardiovascular diseases. Med Clin North Am.

[5] Sethi JM, Rochester CL (2000). Smoking and chronic obstructive pulmonary disease. Clin Chest Med.

[6] Newcomb PA, Carbone PP (1992). The health consequences of smoking. Cancer Med Clin North Am.

[7] Samet JM (2016). Epidemiology and the tobacco epidemic: how research on tobacco and health shaped epidemiology. Am J Epidemiol.

[8] Rigotti NA (2013). Smoking cessation in patients with respiratory disease: existing treatments and future directions. Lancet Respir Med.

[9] Collaborators GBDCRF. The global burden of cancer attributable to risk factors, 2010–19: a systematic analysis for the Global Burden of Disease Study 2019. Lancet. 2022;400(10352):563–91. 10.1016/S0140-6736(22)01438-6 PMC9395583 35988567

[10] Islami F, Marlow EC, Zhao J, Wiese D, Asare S, Bandi P (2022). Person-years of life lost and lost earnings from cigarette smoking-attributable cancer deaths, United States, 2019. Int J Cancer.

[11] Walser T, Cui X, Yanagawa J, Lee JM, Heinrich E, Lee G (2008). Smoking and lung cancer: the role of inflammation. Proc Am Thorac Soc.

[12] Bray F, Ferlay J, Soerjomataram I, Siegel RL, Torre LA, Jemal A (2018). Global cancer statistics 2018: GLOBOCAN estimates of incidence and mortality worldwide for 36 cancers in 185 countries. CA Cancer J Clin.

[13] Hecht SS, Hatsukami DK (2022). Smokeless tobacco and cigarette smoking: chemical mechanisms and cancer prevention. Nat Rev Cancer.

[14] Zito Marino F, Bianco R, Accardo M, Ronchi A, Cozzolino I, Morgillo F (2019). Molecular heterogeneity in lung cancer: from mechanisms of origin to clinical implications. Int J Med Sci.

[15] Hoang PH, Landi MT (2022). DNA methylation in lung cancer: mechanisms and associations with histological subtypes, molecular alterations, and major epidemiological factors. Cancers (Basel).

[16] Muthuramalingam P, Akassh S, Rithiga SB, Prithika S, Gunasekaran R, Shin H (2023). Integrated omics profiling and network pharmacology uncovers the prognostic genes and multi-targeted therapeutic bioactives to combat lung cancer. Eur J Pharmacol.

[17] Zhang Y, Elgizouli M, Schottker B, Holleczek B, Nieters A, Brenner H (2016). Smoking-associated DNA methylation markers predict lung cancer incidence. Clin Epigenet.

[18] Langevin SM, Kratzke RA, Kelsey KT (2015). Epigenetics of lung cancer. Transl Res.

[19] Ordovas JM, Smith CE (2010). Epigenetics and cardiovascular disease. Nat Rev Cardiol.

[20] Sundar IK, Yao H, Rahman I (2013). Oxidative stress and chromatin remodeling in chronic obstructive pulmonary disease and smoking-related diseases. Antioxid Redox Signal.

[21] Herceg Z (2016). Epigenetic mechanisms as an interface between the environment and genome. Adv Exp Med Biol.

[22] Ma YL, Li MD (2017). Establishment of a strong link between smoking and cancer pathogenesis through DNA methylation analysis. Sci Rep.

[23] Ambros V (2004). The functions of animal microRNAs. Nature.

[24] Zhang WC, Liu J, Xu X, Wang G (2013). The role of microRNAs in lung cancer progression. Med Oncol.

[25] da Silva AMG, de Araujo JNG, de Oliveira KM, Novaes AEM, Lopes MB, de Sousa JCV (2018). Circulating miRNAs in acute new-onset atrial fibrillation and their target mRNA network. J Cardiovasc Electrophysiol.

[26] Khan S, Zhang DY, Zhang JY, Hayat MK, Ren J, Nasir S (2022). The key role of microRNAs in initiation and progression of hepatocellular carcinoma. Front Oncol.

[27] Zhang X, Mens MMJ, Abozaid YJ, Bos D, Darwish Murad S, de Knegt RJ (2021). Circulatory microRNAs as potential biomarkers for fatty liver disease: the Rotterdam study. Aliment Pharmacol Ther.

[28] Mens MMJ, Heshmatollah A, Fani L, Ikram MA, Ikram MK, Ghanbari M (2021). Circulatory MicroRNAs as potential biomarkers for stroke risk: the Rotterdam study. Stroke.

[29] Condrat CE, Thompson DC, Barbu MG, Bugnar OL, Boboc A, Cretoiu D (2020). miRNAs as biomarkers in disease: latest findings regarding their role in diagnosis and prognosis. Cells-Basel.

[30] Santos-Alvarez JC, Velazquez-Enriquez JM, Garcia-Carrillo R, Rodriguez-Beas C, Ramirez-Hernandez AA, Reyes-Jimenez E (2022). miRNAs contained in extracellular vesicles cargo contribute to the progression of idiopathic pulmonary fibrosis: an in vitro aproach. Cells-Basel.

[31] Banerjee A, Luettich K (2012). MicroRNAs as potential biomarkers of smoking-related diseases. Biomark Med.

[32] Panico A, Tumolo MR, Leo CG, De Donno A, Grassi T, Bagordo F (2021). The influence of lifestyle factors on miRNA expression and signal pathways: a review. Epigenomics-Uk.

[33] Hijazi K, Malyszko B, Steiling K, Xiao XH, Liu G, Alekseyev YO (2019). Tobacco-related alterations in airway gene expression are rapidly reversed within weeks following smoking-cessation. Sci Rep-Uk.

[34] Beane J, Sebastiani P, Liu G, Brody JS, Lenburg ME, Spira A (2007). Reversible and permanent effects of tobacco smoke exposure on airway epithelial gene expression. Genome Biol.

[35] Wang GQ, Wang R, Strulovici-Barel Y, Salit J, Staudt MR, Ahmed J, et al. Persistence of smoking-induced dysregulation of MiRNA expression in the small airway epithelium despite smoking cessation. PLoS ONE. 2015;10(4). 10.1371/journal.pone.0120824 PMC4401720 25886353

[36] Ikram MA, Brusselle G, Ghanbari M, Goedegebure A, Ikram MK, Kavousi M (2020). Objectives, design and main findings until 2020 from the Rotterdam study. Eur J Epidemiol.

[37] Abozaid YJ, Zhang X, Mens MMJ, Ahmadizar F, Limpens M, Ikram MA (2022). Plasma circulating microRNAs associated with obesity, body fat distribution, and fat mass: the Rotterdam study. Int J Obes (Lond).

[38] Carreras-Torres R, Johansson M, Haycock PC, Relton CL, Smith GD, Brennan P, et al. Role of obesity in smoking behaviour: Mendelian randomisation study in UK Biobank. Bmj-Brit Med J. 2018;361. 10.1136/bmj.k1767 PMC5953237 29769355

[39] Agarwal V, Bell GW, Nam JW, Bartel DP (2015). Predicting effective microRNA target sites in mammalian mRNAs. Elife.

[40] Chen Y, Wang X (2020). miRDB: an online database for prediction of functional microRNA targets. Nucleic Acids Res.

[41] Huang HY, Lin YC, Li J, Huang KY, Shrestha S, Hong HC (2020). miRTarBase 2020: updates to the experimentally validated microRNA-target interaction database. Nucleic Acids Res.

[42] Liu M, Jiang Y, Wedow R, Li Y, Brazel DM, Chen F (2019). Association studies of up to 1.2 million individuals yield new insights into the genetic etiology of tobacco and alcohol use. Nat Genet.

[43] Joehanes R, Just AC, Marioni RE, Pilling LC, Reynolds LM, Mandaviya PR (2016). Epigenetic signatures of cigarette smoking. Circ Cardiovasc Genet.

[44] Kehl T, Kern F, Backes C, Fehlmann T, Stockel D, Meese E (2020). miRPathDB 2.0: a novel release of the miRNA pathway dictionary database. Nucleic Acids Res.

[45] Wixon J, Kell D (2000). The Kyoto encyclopedia of genes and genomes–KEGG. Yeast.

[46] Takahashi K, Yokota S, Tatsumi N, Fukami T, Yokoi T, Nakajima M (2013). Cigarette smoking substantially alters plasma microRNA profiles in healthy subjects. Toxicol Appl Pharm.

[47] Willinger CM, Rong J, Tanriverdi K, Courchesne PL, Huan TX, Wasserman GA (2017). MicroRNA signature of cigarette smoking and evidence for a putative causal role of MicroRNAs in smoking-related inflammation and target organ damage. Circ-Cardiovasc Gene.

[48] Choo KB, Soon YL, Nguyen PN, Hiew MS, Huang CJ (2014). MicroRNA-5p and -3p co-expression and cross-targeting in colon cancer cells. J Biomed Sci.

[49] Huang CJ, Nguyen PN, Choo KB, Sugii S, Wee K, Cheong SK (2014). Frequent co-expression of miRNA-5p and -3p species and cross-targeting in induced pluripotent stem cells. Int J Med Sci.

[50] Keller A, Ludwig N, Fehlmann T, Kahraman M, Backes C, Kern F (2019). Low miR-150–5p and miR-320b expression predicts reduced survival of COPD patients. Cells-Basel.

[51] Velasco-Torres Y, Lopez VR, Perez-Bautista O, Buendia-Roldan I, Ramirez-Venegas A, Perez-Ramos J, et al. miR-34a in serum is involved in mild-to-moderate COPD in women exposed to biomass smoke. Bmc Pulm Med. 2019;19(1). 10.1186/s12890-019-0977-5 PMC6882367 31775690

[52] O'Farrell HE, Bowman RV, Fong KM, Yang IAA (2021). Plasma extracellular vesicle miRNAs can identify lung cancer, current smoking status, and stable COPD. Int J Mol Sci.

[53] Molina-Pinelo S, Pastor MD, Suarez R, Romero-Romero B, De la Gonzalez PM, Salinas A (2014). MicroRNA clusters: dysregulation in lung adenocarcinoma and COPD. Eur Respir J.

[54] Wang WH, Chen J, Zhao F, Zhang BR, Yu HS, Jin HY (2014). MiR-150-5p suppresses colorectal cancer cell migration and invasion through targeting MUC4. Asian Pac J Cancer Prev.

[55] Chen X, Zeng K, Xu M, Hu X, Liu X, Xu T (2018). SP1-induced lncRNA-ZFAS1 contributes to colorectal cancer progression via the miR-150-5p/VEGFA axis. Cell Death Dis.

[56] Yu J, Feng Y, Wang Y, An R (2018). Aryl hydrocarbon receptor enhances the expression of miR-150-5p to suppress in prostate cancer progression by regulating MAP3K12. Arch Biochem Biophys.

[57] Valera VA, Parra-Medina R, Walter BA, Pinto P, Merino MJ (2020). microRNA expression profiling in young prostate cancer patients. J Cancer.

[58] Sugita BM, Rodriguez Y, Fonseca AS, Souza EN, Kallakury B, Cavalli IJ (2022). MiR-150–5p overexpression in triple-negative breast cancer contributes to the in vitro aggressiveness of this breast cancer subtype. Cancers.

[59] Jia HY, Wu D, Zhang ZR, Li SJ (2021). Regulatory effect of the MAFG-AS1/miR-150-5p/MYB axis on the proliferation and migration of breast cancer cells. Int J Oncol.

[60] Xiao GD, Wang PL, Zheng XQ, Liu DP, Sun X (2019). FAM83A-AS1 promotes lung adenocarcinoma cell migration and invasion by targeting miR-150-5p and modifying MMP14. Cell Cycle.

[61] Chen F, Wang X, Jang SK, Quach BC, Weissenkampen JD, Khunsriraksakul C (2023). Multi-ancestry transcriptome-wide association analyses yield insights into tobacco use biology and drug repurposing. Nat Genet.

[62] Godoy PM, Barczak AJ, DeHoff P, Srinivasan S, Etheridge A, Galas D (2019). Comparison of reproducibility, accuracy, sensitivity, and specificity of miRNA quantification platforms. Cell Rep.

[63] de Bruijn RF, Bos MJ, Portegies ML, Hofman A, Franco OH, Koudstaal PJ (2015). The potential for prevention of dementia across two decades: the prospective, population-based Rotterdam study. BMC Med.

[64] Sticht C, De La Torre C, Parveen A, Gretz N (2018). miRWalk: an online resource for prediction of microRNA binding sites. PLoS ONE.

