# Supplementary material for: Smoking-related dysregulation of plasma circulating microRNAs: the Rotterdam study
Source: Hum Genomics. 2023 Jul 10;17:61. doi: 10.1186/s40246-023-00504-5 (PMC10331979; doi:10.1186/s40246-023-00504-5)
Supplement: Supplementary file 2 — Additional file 2: Fig. S1. Provides the distribution of the top 10 significantly associated miRNAs with current versus never smoking status; Fig. S2. Provides a Volcano plot depicting the results from current versus former smokers analysis, where current smoking was a reference; Fig. S3. Depicts boxplots of all 41 smoking miRNAs with their expression levels across different smoking cessation categories, while Fig. S4. Depicts the enrichment plot for the smoking associated miRNAs in the KEGG pathways. [file 40246_2023_504_MOESM2_ESM.docx]

**Additional file 2: Supplementary Figures**

**Figure S1. Distribution of the top 10 miRNAs associated with current (n=382) versus never (n=921) smoking status**

**Figure S2. MicroRNA expression levels associated with tobacco smoking in current (reference)] versus former smokers**

**Figure S3. Expression levels of the smoking-miRNAs across different categories of smoking cessation**

**Figure S4. KEGG pathways depicting the pathways regulated by smoking-associated miRNAs (current versus never[reference])**
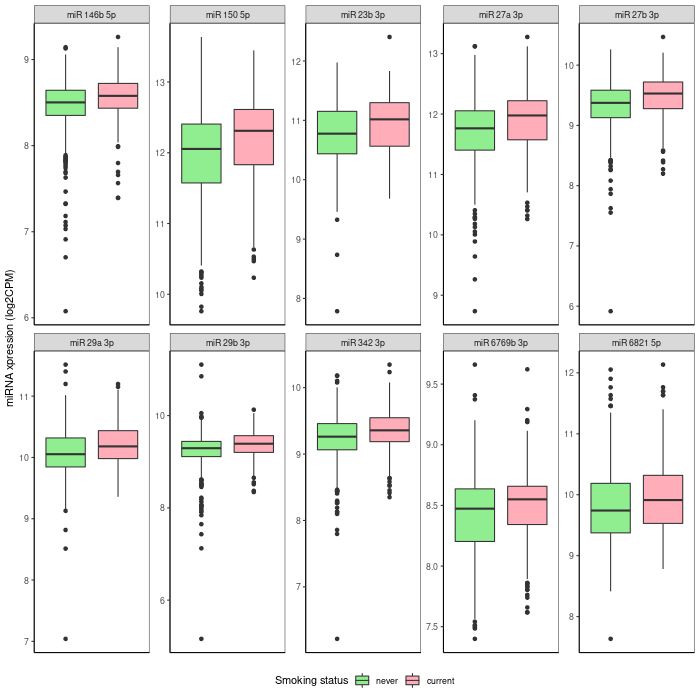
**Figure S1. Distribution of the top 10 miRNAs associated with current (n=382) versus never (n=921) smoking status**

Ten boxplots showing the distribution of miRNA levels between current and never smokers for the top 10 miRNAs. The X-axis depicts the smoking status (current versus never smokers), while Y-axis depicts plasma miRNA levels in log2 CPM. The red color indicates current smokers, while green indicates never smokers. Abbreviations: miRNAs: microRNAs, CPM: counts per million.

**
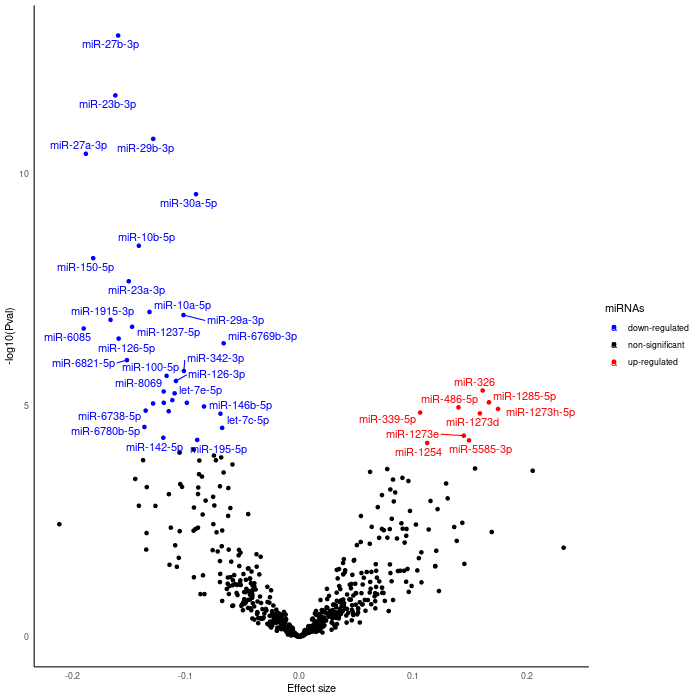
Figure S2. MicroRNA expression levels associated with tobacco smoking in current (reference) versus former smokers**

This Volcano plot shows the 591 well-expressed miRNAs as dots in the association of current versus former smoking status, where current is a reference using a linear regression model. Blue color depicts negatively associated miRNAs with smoking status, while red color depicts positively associated miRNAs with smoking status. The effect size per miRNA in the analysis is reflected on the x-axis, while the magnitude of significance is shown on the y-axis. Bonferroni correction threshold of P<0.05/591=8.46×10^-5^ was implemented.


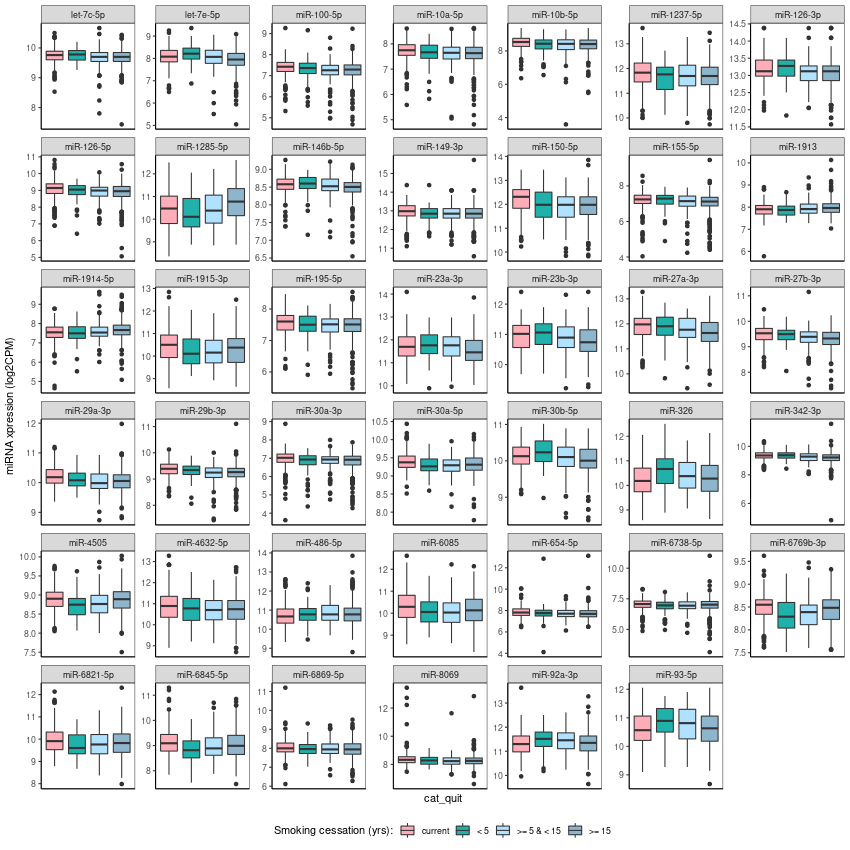
**Figure S3. Expression levels of the smoking-miRNAsacross different categories of smoking cessation**

The figure depicts the smoking- miRNAs and their respective expression levels across smoking cessation categories (years since quitting) on X-axis, while Y-axis represents miRNA expression levels. Smoking groups are divided as follow: current smokers (n=382), <5 years (n=83), ≥5 and <15 years (n=161), and ≥15 years (n=535) cessation time.

**Figure S4. KEGG pathways depicting the pathways regulated by smoking-associated miRNAs (current versus
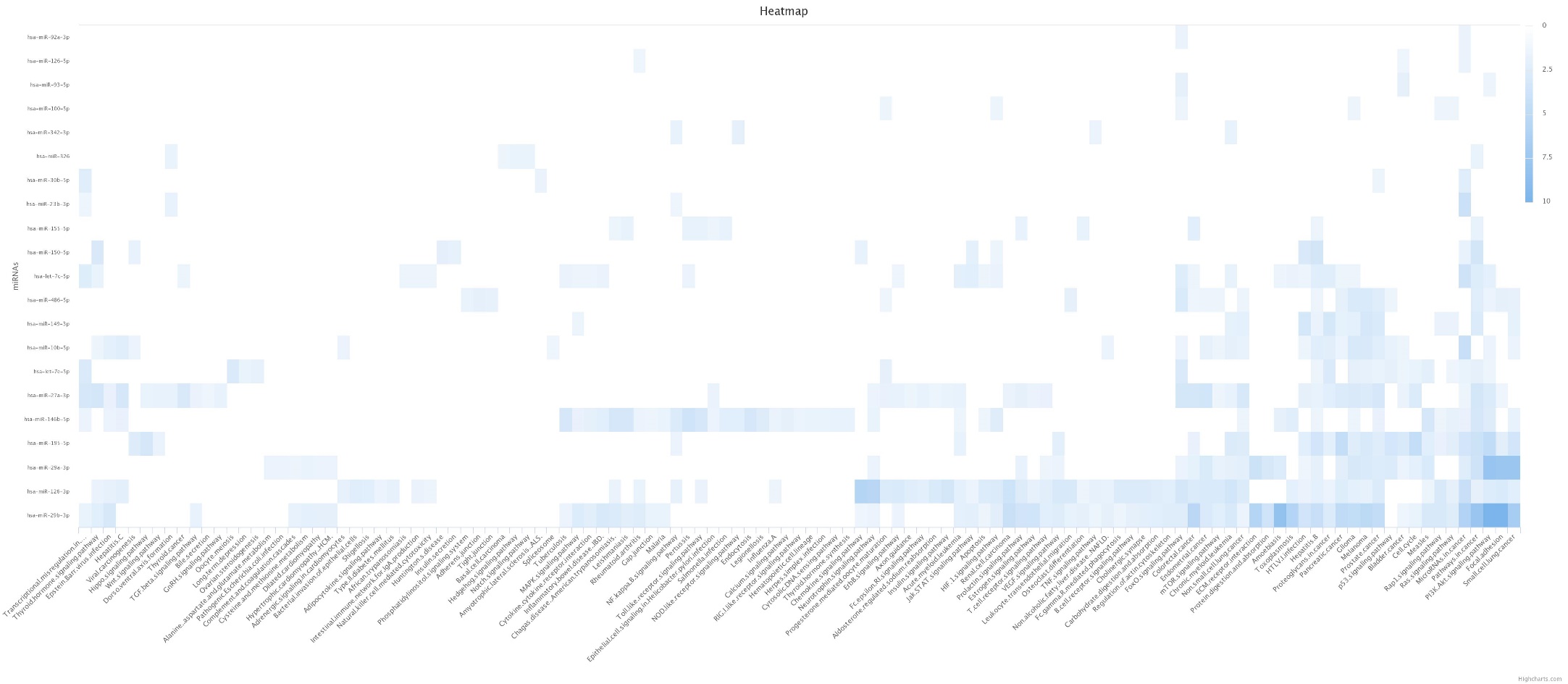
never[reference])**

The heatmap depicts enrichment results regulated by smoking-associated miRNAs (current versus never) for the categories within KEGG database and experimentally validated miRNA-target interactions. Columns depicts all KEGG pathways significant for the different miRNAs, while rows illustrate enrichment results for the target genes of smoking-associated miRNAs. The blue color represents the –log10 transformed P-value of the enrichment results, the darker the color- the more significant the association between a given miRNA and the target pathway.
